# Effect of vestibular loss on head-on-trunk stability in individuals with vestibular schwannoma

**DOI:** 10.1038/s41598-024-53512-3

**Published:** 2024-02-12

**Authors:** Raabeae Aryan, Omid A. Zobeiri, Jennifer L. Millar, Michael C. Schubert, Kathleen E. Cullen

**Affiliations:** 1 Department of Biomedical Engineering, Johns Hopkins University School of Medicine, 720 Rutland Ave, Traylor 504, Baltimore, MD 21205-2109 USA; 2 Department of Biomedical Engineering, McGill University, Montreal, QC Canada; 3 Department of Physical Medicine and Rehabilitation, Johns Hopkins University School of Medicine, Baltimore, MD USA; 4 Department of Otolaryngology-Head and Neck Surgery, Johns Hopkins University School of Medicine, Baltimore, USA; 5 Department of Neuroscience, Johns Hopkins University School of Medicine, Baltimore, USA; 6 Kavli Neuroscience Discovery Institute, Johns Hopkins University, Baltimore, MD USA

**Keywords:** Inner ear, Rehabilitation

## Abstract

The vestibulo-collic reflex generates neck motor commands to produce head-on-trunk movements that are essential for stabilizing the head relative to space. Here we examined the effects of vestibular loss on head-on-trunk kinematics during voluntary behavior. Head and trunk movements were measured in individuals with vestibular schwannoma before and then 6 weeks after unilateral vestibular deafferentation via surgical resection of the tumor. Movements were recorded in 6 dimensions (i.e., 3 axes of rotation and 3 axes of translation) using small light-weight inertial measurement units while participants performed balance and gait tasks. Kinematic measures differed between individuals with vestibular schwannoma (at both time points) and healthy controls for the more challenging exercises, namely those performed in tandem position or on an unstable surface without visual input. Quantitative assessment of the vestibulo-ocular reflex (VOR) revealed a reduction in VOR gain for individuals with vestibular schwannoma compared to control subjects, that was further reduced following surgery. These findings indicated that the impairment caused by either the tumor or subsequent surgical tumor resection altered head-on-trunk kinematics in a manner that is not normalized by central compensation. In contrast, we further found that head-on-trunk kinematics in individuals with vestibular schwannoma were actually comparable before and after surgery. Thus, taken together, our results indicate that vestibular loss impacts head-on-trunk kinematics during voluntary balance and gait behaviors, and suggest that the neural mechanisms mediating adaptation alter the motion strategies even before surgery in a manner that may be maladaptive for long-term compensation.

## Introduction

The vestibular system detects the head motion experienced during our daily activities. In turn, vestibulo-spinal pathways generate motor responses to ensure the maintenance of balance and accurate control of posture^1–3^. One particularly well studied vestibulo-spinal reflex pathway is the vestibulo-collic reflex (VCR), which generates head-on-trunk motion to stabilize the head relative to space^4,5^, where trunk motion is that of the upper portion of the body comprising the waist and neck. In its most direct form, the vestibulo-collic reflex is mediated by a direct 3-neuron arc, comprising projections from the vestibular afferents of the VIIIth nerve to the vestibular nuclei. In turn, these vestibular nuclei neurons target neck motoneurons via descending projections to the spinal cord^4^. Additionally, indirect pathways involving projections from relay structures such as the interstitial nucleus of Cajal and reticular formation to the spinal cord make major contributions to this reflex^4^.

The vestibulo-collic reflex is thought to play an essential role in gaze stabilization and postural control during everyday life. For example, minimal head pitch movement occurs relative to space during walking because head pitch relative to the trunk is largely compensatory for trunk pitch^6^. Overall, it is generally agreed that such head stabilization is achieved via the combined effects of vestibulo-collic reflex and biomechanics of head motion, with the former making the most significant contribution for frequencies below 2 Hz^7,8^, corresponding to the natural frequency range of walking^9,10^. Furthermore, individuals with bilateral peripheral vestibular loss demonstrate impaired head-on-trunk stabilization in pitch and roll planes during upright standing compared to controls^12^. To date, however, how head-on-trunk kinematics are altered as a result of peripheral vestibular loss during voluntary behaviors such as walking or challenging balance exercises is not well understood. Crane and Demer measured head and trunk stability during treadmill walking and running in individuals with unilateral vestibular loss, yet they did not quantify head-on-trunk kinematics in this group of people^13^. Furthermore, while two more recent studies characterized head-on-trunk movements in individuals with complete unilateral vestibular loss^14,15^, these studies solely focused on voluntary head rotations (i.e., yaw rotations) and only quantified motion in a single dimension.

Accordingly, here we sought to explore whether head-on-trunk kinematics are altered due to peripheral vestibular loss during voluntary balance and gait behaviors. We focused on individuals with vestibular schwannoma (VS)—a tumor that grows slowly due to the overproduction of the Schwann cells of the VIII cranial nerve. Surgical removal of the tumor is one of the therapeutic options for managing VS that necessarily ablates the vestibular nerve on the affected side. We hypothesized that kinematics of head-on-trunk stabilization in individuals with VS, both before or after the VS surgery, will differ from those of healthy controls. Additionally, we hypothesized that kinematics of head stabilization will show more impairment following VS surgery as compared to before surgery. Head-on-trunk movements were characterized while participants performed a series of commonly prescribed balance and gait exercises before and following (6 weeks) surgical resection of the tumor. We did this by first recording head and trunk movements in all six dimensions of motion (three rotational, three translational axes) using two inertial measurement sensors (IMUs). We then computed head motion relative to trunk motion and quantified its kinematics by computing the range of motion and measures of variability for each axis. Overall, we found that head-on-trunk kinematics during the most challenging exercises (i.e., those tasks performed in tandem position or on an unstable surface without visual input) differentiated both pre-operative and post-operative individuals with VS from the age-matched healthy controls. Notably, during these tasks, individuals with VS displayed obvious changes in the range and variability of rotational head kinematics, particularly along the yaw as well as the roll axes. Taken together our results show that head-on-trunk kinematics are significantly altered in individuals with VS, even prior to surgery as a result of the presence of the tumor, suggesting that early central compensation sets an upper limit for recovery. Thus, our results support the need to advance the development of more clinically relevant pre- and post-operative interventions through focusing on tests and rehabilitative strategies that improve roll and yaw head control in individuals with VS.

## Methods

### Participants

Eighteen individuals with unilateral VS who were able to ambulate independently and were scheduled for tumor resection via suboccipital craniotomy were recruited into this study; from which, 9 male participants (mean age = 56.1 ± 15.7 years old, range 24–73 years old) were able to complete both pre-operative (mean = 8 ± 13 days) and 6 weeks post-operative assessments (36–42 days). Patients were excluded if they were unavailable for the 6 weeks post-operative testing, experienced post-operative changes including unexpected infection (meningitis), post-operative mass effect of brainstem or cerebellum, or pathology report ended up being a meningioma rather than VS. Pre-operative complaints of individuals with VS were as follows: 78% complained of auditory impairment with 100% having documented sensorineural hearing loss via audiogram, dizziness (33%), imbalance (33%), tinnitus (44%), pain (44%), headache (22%), ear fullness (22%), and facial involvement (56%) (see supplementary Tables S2 and S3 for more details about the VS group’s pre-operative characteristics including tumor size, and laterality). None of our VS participants received pre-operative gentamicin treatment. Additionally, 9 age-matched healthy controls without a history of neurologic or otologic conditions were recruited (8 male and 1 female participants, mean age = 49.3 ± 15.0 years old, range 24–72 years old). The kinematic tests of gait and balance, and clinical characteristics of participants were assessed concurrently. This study was approved by the Johns Hopkins Institutional Review Board and performed in accordance with the institution’s guidelines for safe and ethical research in human subjects. Written informed consent forms were obtained from both VS and control groups before the data collection.

### Clinical characteristics

Video Head Impulse Test (vHIT; ICS Otometrics, Natus Medical Incorporated, Denmark) was used to measure the gain of vestibulo-ocular reflex (VOR) during passive head rotations along all 3 semicircular canal planes for each ear^24,25^. Dynamic Visual Acuity test (DVA)^16^ was used to identify efficacy of the VOR reflex during active sinusoidal head rotations as previously described^17^. Additionally, the 10-m walk test with normal speed^18^, Timed up and Go test (TUG)^19–21^, and the Functional Gait Assessment scale (FGA)^22,23^ were assessed in both healthy controls and pre- and post-operative individuals with VS. Participants reported their self-perceived balance confidence, and impact of headache, anxiety, and dizziness on their daily life functioning by using respectively Activities-Specific Balance Confidence scale (ABC)^26^, Headache Impact Test (HIT-6)^27^, Beck Anxiety Inventory (BAI)^28^, and Dizziness Handicap Inventory (DHI) questionnaires^29^.

### Balance and gait exercises

Participants performed balance and gait exercises (Table 1) while wearing inertial measurement units (IMUs) affixed on their heads and trunks. Specifically, we focused on 7 standing balance tasks performed during two visual conditions (i.e., eyes open or closed) and across varied proprioceptive conditions (i.e., firm versus unstable surfaces, standard versus tandem (heel to toe) base of supports). In addition, we quantified motion during 3 tandem walking tasks (Table 1), including the 7th item of the FGA scale (i.e., tandem walk forward for 10 steps), as well as during two extended-duration tandem walk tasks in which the participants walked forward and backward for 30 s. In general, if individuals lost their balance in the middle of a test, participants were instructed to regain their balance and return to the test position (if possible) and continue until the 30 s expired.

**Table 1:** List of 10 balance and gait tasks performed in this study.

Tasks		Duration	Visual condition	Base of support
1	Tandem walk forward (FGA item #7)	10 steps	EO	Tandem
2	Tandem walk forward	30 s	EO	Tandem
3	Tandem walk backward	30 s	EO	Tandem
4	Tandem stance	30 s	EO	Tandem
5	Tandem stance	30 s	EC	Tandem
6	Standing on firm surface	30 s	EC	Firm
7	Standing on foam	30 s	EC	Unstable
8	Standing on foam	30 s	EO	Unstable
9	Foam cup balance, one foot	30 s	EO	Unstable
10	Foam cup balance, alternate feet	30 s	EO	Unstable

*FGA* Functional Gait Assessment, *EO* eyes-open condition, *EC* eyes-closed condition.

### Quantification of kinematics

We recorded the 6-dimensional head and trunk motions of each participant using 2 IMUs (Shimmer3 IMU, Shimmer Research, Dublin, Ireland), which were attached to the back of participants’ head, and trunk approximately between the L4-S1 vertebrae. Prior to affixing the sensors on participants, data collection in the two sensors were synchronized using the ConsensysPro software (Shimmer Research, Dublin, Ireland). Kinematic data from each sensor (i.e., 3-dimensional linear acceleration (translational) along the anterior–posterior, medial–lateral, and vertical axes, as well as 3-dimensional rotational velocity (angular) in the yaw, pitch, and roll planes) were sampled at 500 Hz, and saved on the IMUs’ built-in micro-SD cards for off-line processing.

**Figure 1:**
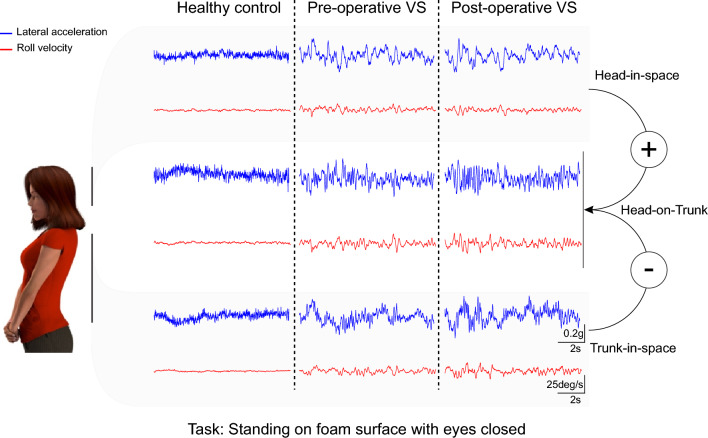
Example lateral linear acceleration (blue) and roll rotational velocity (red) of head-in-space (top row), head-on-trunk (middle row), and trunk-in-space (bottom row). The presented data are from a typical healthy control (left column), and one vestibular schwannoma participant pre-operatively and post-operatively (center and right columns, respectively) recorded during “standing on foam with eyes closed” exercise. VS: vestibular schwannoma.

To quantify the kinematics of head-on-trunk during each exercise in Table 1, we first computed 6-dimensional head-on-trunk motion by comparing motion of the head and trunk in each dimension and calculating the difference (Fig. 1) for healthy controls and individuals with VS both pre- and 6 weeks post-operatively. We then computed the total root mean square (RMS), range, and standard deviation (SD) of head motion, and resulting head-on-trunk data for each axis. All analyses focused on segments of recordings during which participants were able to control their spatial orientation without losing balance.

### Statistics and computation of global kinematic score

To investigate whether head-on-trunk kinematics were altered due to peripheral vestibular loss during voluntary behaviors, we compared these measures in healthy controls and individuals with vestibular schwannoma. Comparison analyses between healthy controls and vestibular schwannoma groups were performed by using a non-parametric paired sample permutation (re-randomization) test. We generated 2000 randomized rearrangements of the observed data points and then computed p-values of the actual observed measures. To find the consistent trends across several exercises, we assessed whether the correlation for the majority of exercises a) were significant (*p* < 0.05, Pearson correlation), and b) had the same sign (i.e., correlation was consistently positive/negative across exercises). Correction for multiple comparisons was not performed since the goal of this exploratory study was to investigate individuals with unilateral vestibular loss already known to be different from healthy controls based on clinical assessment and thus performing correction would have exaggerated Type II errors. Statistical significance level was set at α = 0.05; and values reported as mean ± 1 SD. Statistical and data processing were performed using custom written MATLAB routines (The MathWorks, Inc., Natick, MA, USA).

Additionally, we computed a single kinematic score according to the average weighted linear combination of all kinematic measures. To do this, each computed kinematic measure was normalized by a linear transformation of mean ± 2SD to obtain a number between 0 and 100 (i.e., normalized mean = 50 and normalized SD = 25). Values outside the 0–100 range were then projected to the closest value in this range (i.e., to 0 or 100). This score was computed for the 2 most informative exercises for which the most significant differences were observed between the individuals with VS and healthy controls, namely “tandem stance with eyes closed” and “standing on foam with eyes closed”, as well as for these exercises and “standing on a firm surface with eyes closed”.

## Results

**Figure 2:**
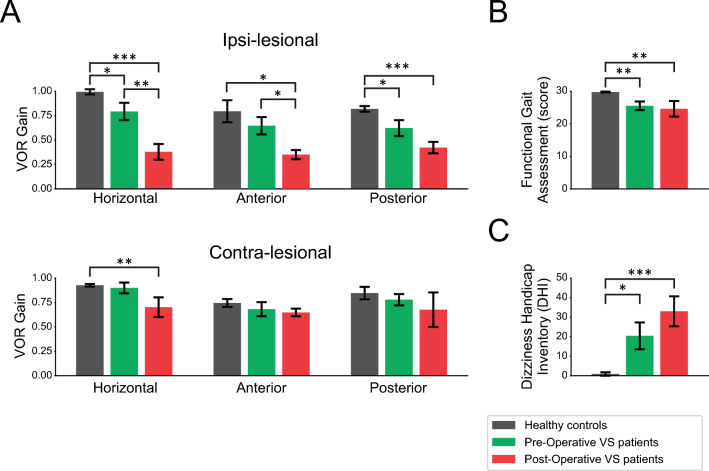
Between groups comparison of (**A**) Ipsi-lesional vHIT VOR gains (top) and contra-lesional (bottom) vHIT VOR gains for all 3 canals; (**B)** Functional Gait Assessment score; and (**C)** Dizziness Handicap Inventory; (p-value: * < 0.05, ** < 0.01, *** < 0.001). VOR: vestibulo-ocular reflex. VS: vestibular schwannoma.

We first assessed vestibular function and functional balance using VOR testing and standard scoring on the FGA (described in Table 2). Figure 2A illustrates the distribution of VOR gains measured using the video Head Impulse Test (vHIT, see Methods) for healthy controls, and pre-operative and then post-operative individuals with VS. Comparison across populations revealed that horizontal vHIT gains were significantly reduced in post-operative individuals with VS, relative to controls, for both ipsi-lesional and contra-lesional testing (*p* < 0.001, and *p* < 0.01, respectively). In addition, post-operative individuals with VS demonstrated significantly decreased gains for anterior and posterior vHIT testing, relative to controls for ipsi-lesional testing (*p* < 0.05, and *p* < 0.001, respectively). Prior to surgery, individuals with VS also showed a significant decrease in horizontal and posterior vHIT gains relative to controls for ipsi-lesional testing (*p* < 0.05). In contrast, analysis of vHIT gains for contral-lesional testing revealed no difference between healthy controls and pre-operative individuals with VS (*p* > 0.05). Following surgery, post-operative individuals with VS further showed a significant decrease in ipsi-lesional horizontal and anterior vHIT gains in comparison to the pre-operative time point, but no difference in their ipsi-lesional posterior vHIT gains (*p* > 0.05).

**Table 2:** Mean (SD) of clinical measures for all groups.

Clinical Measures	Healthy Control	Pre-operative VS	Post-operative VS
Lesion side (right/left)	*None*	6/3	6/3
**Functional measures**			
FGA Scale (score, max 30)	29.8 (0.4)*^₸^	25.6 (3.9)	24.6 (6.8)
TUG with ipsi-lesional turn (sec)	6.3 (1.3)^₸^	7.7 (3.0)	9.8 (6.2)
TUG with contra-lesional turn (sec)	6.4 (1.1)	7.5 (2.8)	9.3 (5.7)
Normal gait speed (m/sec)	1.5 (0.2)^₸^	1.4 (0.3)	1.2 (0.3)
DVA LogMar (score)			
*Static*	-0.12 (0.1)	-0.04 (0.2)	-0.07 (0.1)
*Corrected ipsi-lesional*	0.23 (0.1)^₸^	0.36 (0.2)	0.52 (0.3)
*Corrected contra-lesional*	0.21 (0.1)^₸^	0.30 (0.1)^₸^	0.60 (0.3)
**Physiological measures**			
Ipsi-lesional VOR gain			
*Horizontal*	0.99 (0.1)*^₸^	0.79 (0.3)^₸^	0.38 (0.3)
*Anterior*	0.80 (0.3)^₸^	0.65 (0.3)^₸^	0.35 (0.2)
*Posterior*	0.82 (0.1)*^₸^	0.62 (0.2)	0.42 (0.2)
Contra-lesional VOR gain			
*Horizontal*	0.93 (0.0)^₸^	0.90 (0.2)	0.70 (0.4)
*Anterior*	0.75 (0.1)	0.68 (0.2)	0.65 (0.3)
*Posterior*	0.85 (0.2)	0.78 (0.2)	0.68 (0.5)
**Subjective measures**			
Dizziness Handicap Inventory (max 100)	0.9 (2.8)*^₸^	20.4 (21.8)	33.0 (24.7)
Activities-specific balance confidence (%)	94.4 (7.4)^₸^	86.1 (17.7)	73.6 (27.4)
Beck anxiety inventory (max 63)	2.8 (3.1)^₸^	7.6 (6.0)	7.0 (4.5)
Headache impact test score (max 78)	37.0 (15.8)*^₸^	50.2 (7.4)	52.1 (10.4)

*Significant difference with Pre-op group. ₸ Significant difference with Post-op group.

VS, vestibular schwannoma; FGA, Functional Gait Assessment; TUG, Timed and Up Go test; DVA, dynamic visual acuity test; VOR, vestibulo-ocular reflex.

*For healthy controls, right was the ipsi-lateral side, and left was the contra-lateral side.

Each participant's functional capacity was also scored using the conventional FGA test evaluated by a clinician (an integer value between 0 and 3 was assigned to each of the 10 tasks). Figure 2B demonstrates that the FGA scores of individuals with VS were significantly worse than those of healthy controls at each of our 2 time points (i.e., pre-operatively and 6 weeks post-operatively; *p* < 0.01). We further found that FGA scores did not differ for individuals with VS when compared across these 2 time points (*p* > 0.05). Specifically, the FGA scores of individuals with VS remained unchanged at the 6-week post-operative time point relative to their pre-operative scores. Finally, we further compared the results of VS participants and healthy controls on a 25-item self-report questionnaire that quantifies the impact of dizziness on daily life by measuring self-perceived handicap (i.e., the Dizziness Handicap Inventory (DHI, see Methods). As shown in Fig. 2C**,** DHI scale scores were significantly higher in individuals with VS when compared to healthy controls consistent with greater dizziness in the former group. This difference with the control group was most prominent at the post-operative time point (*p* < 0.001); however no significant difference in DHI score was observed between pre- and post-operative time points (*p* > 0.05).

### Head-on-trunk kinematics are significantly different between the healthy controls and pre- and post-operative individuals with vestibular schwannoma

**Figure 3:**
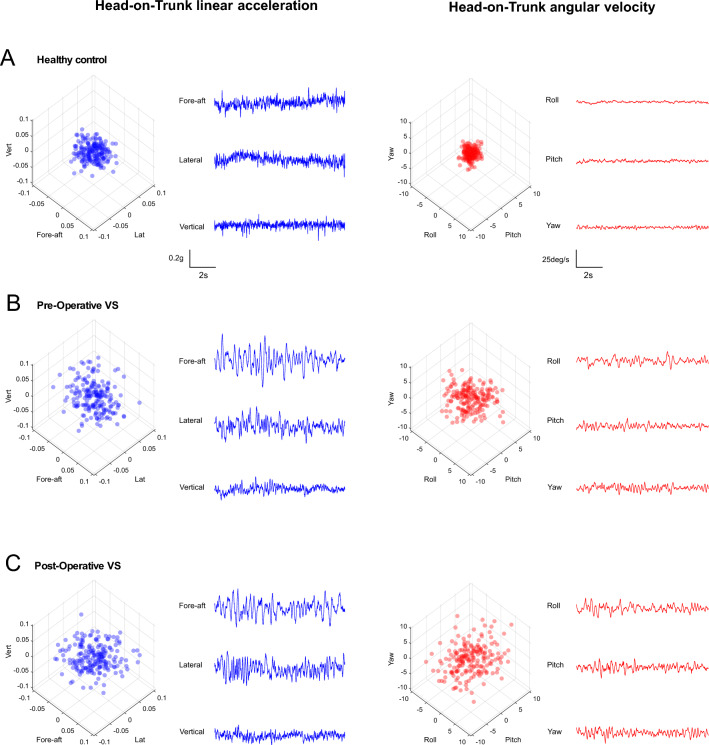
Example head-on-trunk kinematic data from a typical (**A**) healthy control, and (**B,C**) a vestibular schwannoma participant pre-operatively and post-operatively, respectively. Data were collected during the “standing on foam with eyes closed” exercise during which participants were asked to stand on a rectangular foam (39 × 32 × 6 cm^3^) with their feet together and their eyes closed. The blue 3D scatter plots and time series show the head-on-trunk linear acceleration in 3 axes of translation (fore-aft, lateral, and vertical). The red 3D scatter plots and time series show the head-on-trunk angular velocity in 3 axes of rotation (roll, pitch, and yaw). Note the 100 × scaling difference in axes.

**Figure 4:**
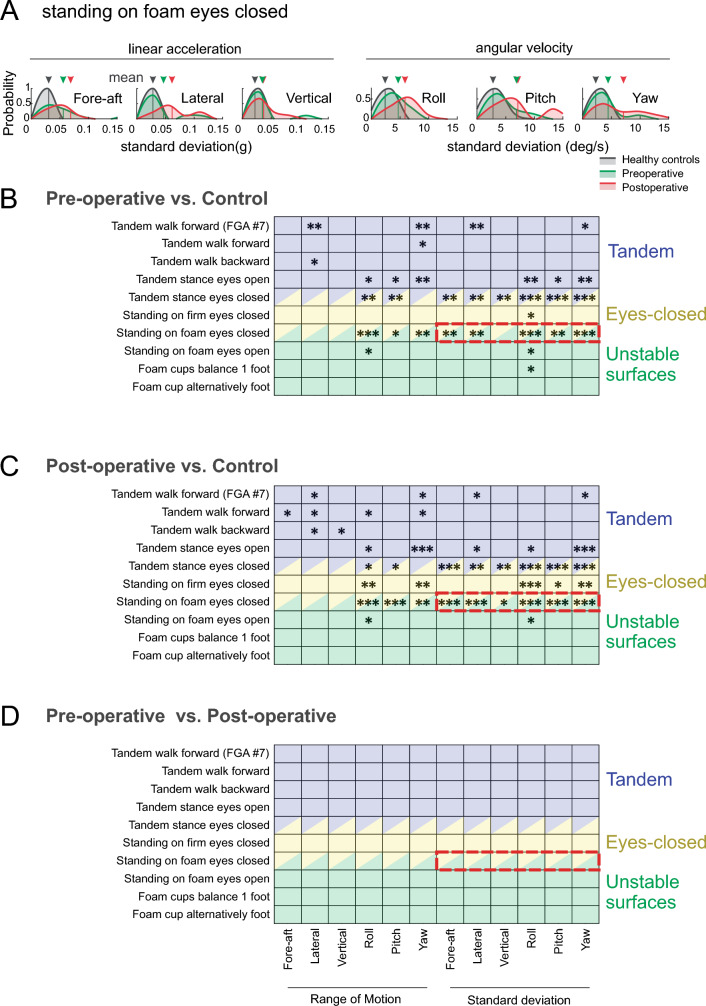
(**A**) The distribution of the standard deviation of head-on-trunk motion in all six axes during “standing on foam with eyes closed” for healthy controls and individuals with VS. Comparison of kinematic variables of the head-on-trunk motion between (**B**) pre-operative vestibular schwannoma group and healthy controls, (**C**) post-operative vestibular schwannoma group and healthy controls, and (**D**) pre-operative and post-operative vestibular schwannoma groups. The kinematic variables are arranged horizontally along the x-axes. The 10 balance exercises are arranged vertically (y-axes) and categorized into 3 groups: tandem (purple), eyes-closed (yellow), and unstable surface (green). Rows shaded in purple-yellow, and green-yellow denote tandem stance with eyes closed, and standing on foam with eyes closed, respectively. Asterisks indicate significant differences between the two groups (*p*-value: * < 0.05, ** < 0.01, and *** < 0.001).

Overall, individuals with VS displayed a greater head-on-trunk variability compared to the healthy controls that was indicative of their unstable head motion during this exercise. Figure 3A–C show the increased variability in the head-on-trunk motion generated by a typical VS participant either pre- or post-operatively (Fig. 3B,C, respectively), versus that generated by a typical healthy control participant (Fig. 3A), during one of the most challenging exercises: standing on foam with eyes closed (Table 1, exercise #7). The blue and red 3D scatter plots provide a 3D representation of linear and rotational head motion, respectively. The distributions in Fig. 4A compare the variability across all participants during this same exercise. Notably, the variability of head-on-trunk motion in individuals with VS (both pre- and post-operatively) was higher in all six linear (Fig. 4A, left) and rotational axes (Fig. 4A, right) than that of healthy controls.

We then further investigated whether there were additional differences in the head-on-trunk kinematics of healthy controls versus individuals with VS. Figure 4B–D illustrate the differences in measures obtained from our quantification of head-on-trunk motion kinematics (see Methods) for our populations of controls versus individuals with VS, during each of the 7 balance and 3 gait exercises. Exercises were grouped into three categories: (1) ‘tandem exercises’ during which the toes of the backward foot touch the heel of the forward foot (purple shaded block), (2) ‘eyes-closed exercises’ during which participants did not have visual input (yellow shaded block), and (3) exercises that required standing on an ‘unstable surface’ (green shaded block).

Figure 4B compares the head-on-trunk kinematic measures for pre-operative VS participants versus healthy controls during each of the 10 exercises, and shows which of the kinematic measures and tasks were significantly higher for the pre-operative than healthy group. Results suggest that tasks of standing on foam with eye closed and tandem standing with eyes closed, followed by tandem standing with eyes-open seem to be the most challenging tasks for the pre-operative individuals with VS as they reveal the most statistically significant differences in head-on-trunk kinematics between the controls and pre-operative individuals with VS. Furthermore, the differences between the pre-operative individuals with VS and healthy controls are more prominent in the rotational kinematic measures than in the linear measures; that is, the pre-operative group demonstrated mainly greater range and variability (SD) of rotational velocities in yaw, roll, and pitch planes compared to healthy controls (Fig. 4B, *p-value < 0.05, ** < 0.01, *** < 0.001).

Figure 4C shows that in the post-operative group all 3 eye closed balance tasks appeared to be the most challenging tasks, namely tasks of standing on foam with eye closed, tandem standing with eyes closed, and standing on a firm surface with eyes-closed. Again, here the significant differences between healthy controls and post-operative individuals with VS are mainly evident for the range and variability of rotational kinematic measures than the linear measures. Most prominently, in comparison to healthy controls, greater range and variability in rotational velocities respectively in yaw, roll, and pitch planes are observed across multiple tasks in the post-operative group (Fig. 4C, p* < 0.05, ** < 0.01, *** < 0.001). And finally, Fig. 4D shows that there were no significant differences in the kinematics of head-on-trunk movement between the pre-operative and 6 weeks post-operative VS groups during any of the challenging gait and balance exercises (*p* > 0.05).

Thus, overall, we found that relative to the healthy controls, pre-operative and post-operative individuals with VS experience more variable head-on-trunk rotational movements (*p* < 0.05), particularly while performing tandem stance with eyes closed, and standing on a foam surface with eyes closed.

### Pre-operative head-on-trunk kinematics correlate with multiple pre-operative clinical measures in individuals with VS

**Figure 5:**
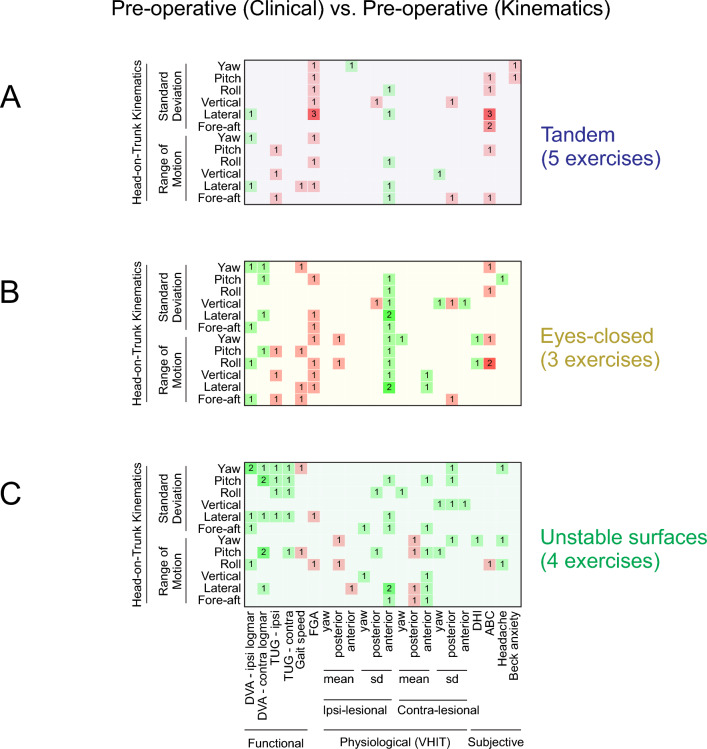
Correlations between pre-operative head-on-trunk kinematic variables and pre-operative clinical measurements for: (**A**) tandem exercises, (**B**) eyes-closed exercises, and (**C**) exercises performed on unstable surfaces. In each panel, the green cells indicate positive correlations, the red cells indicate negative correlations. Brightness and number in the cells denote the number of exercises for which there was a significant correlation (*p*-value < 0.05).

We next asked whether there were any relationships between the pre-operative head-on-trunk kinematics and pre-operative clinical measures in individuals with VS. Figure 5 illustrates the correlations observed between the pre-operative head-on-trunk kinematics and pre-operative functional (i.e., DVA, TUG, gait speed, FGA), physiological (i.e., vHIT mean and SD of VOR gains), and subjective clinical measures (i.e., DHI, ABC, Headache impact, Beck anxiety questionnaires). In this figure, values presented in each cell of the correlational matrices correspond to the number of exercises in which a specific kinematic head-on-trunk measure was significantly correlated with a clinical measure (*p* < 0.05). Overall, our results mainly revealed significant correlations between the variability of kinematic measures and several clinical measures. In particular, we found that poorer functional performance (Fig. 5A–C; e.g., FGA, TUG, DVA LogMar scores) was correlated with higher variability (SD) of head-on-trunk kinematic measures in pre-operative individuals with VS. In addition, physiological measures showed significant correlations with several kinematic measures during balance exercises with eyes closed. Specifically, there were consistent correlations (in 2/3 eyes-closed tasks) between the SD of ipsi-lesional anterior VOR gain and the range and SD of head-on-trunk lateral acceleration (Fig. 5B). Finally, as shown in Figs. 5A,B the pre-operative balance confidence score (ABC, a subjective measure) was consistently inversely correlated with head-on-trunk kinematics, during tasks performed in tandem position (SD of lateral acceleration in 3/5 tasks), and with eyes closed (range of roll velocity in 2/5 tasks). Thus, head-on-trunk kinematics correlated with multiple clinical measures in individuals with VS at the pre-operative time point. For completeness, supplemental Figures S1A,B show the results of correlational analysis between the post-operative kinematic measures and pre- and post-operative clinical measures of individuals with vestibular schwannoma.

### The global change in head-on-trunk kinematics, quantified based on the most informative kinematic parameters, can differentiate vestibular schwannoma groups from healthy controls

**Figure 6:**
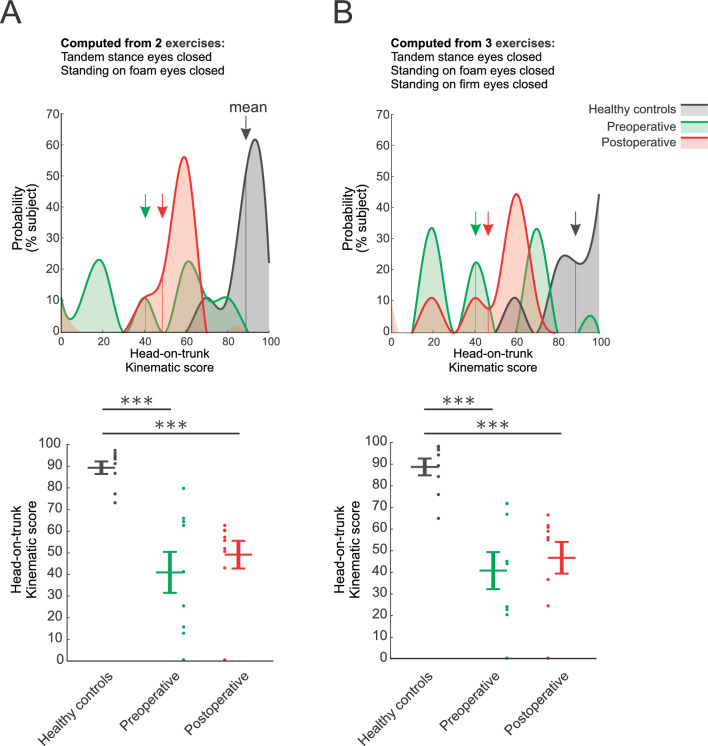
Comparison of global kinematic scores computed from (**A**) the 2 most informative balance exercises "standing on foam eyes-closed", and “Tandem stance with eyes closed”, and (**B**) from all 3 eyes-closed tests. (**A**) and (**B**), top panels: Probability distributions of the kinematic scores computed for healthy controls (black), pre-operative (green), and post-operative vestibular schwannoma groups (red). Arrows indicate the average values. (**A**) and (**B**), bottom panels: Comparison of the kinematic scores of healthy controls versus vestibular schwannoma groups. Vertical lines correspond to mean ± SEM of the kinematic score for each group, while the kinematic score for individual participants is illustrated as points. Asterisks denote significant differences between healthy controls and vestibular schwannoma groups (***p-value < 0.001).

Finally, we assessed whether it was possible to distinguish the performance of subjects in different groups using a reduced number of exercises and kinematic variables. To address this, we computed a single kinematic score (described in “Methods”) that ranged from 0 (most altered) to 100 (comparable to healthy controls). We first used the 2 most informative exercises—those for which the most significant differences were observed between the individuals with VS and healthy controls—namely “tandem stance with eyes closed” and “standing on a foam with eyes closed” (Fig. 6A). Next, we used the 3 most informative exercises which included the same 2 exercises as well as “standing on a firm surface with eyes closed”, illustrated in Fig. 6B. Overall, the scores calculated from both computations were approximately similar, in which the healthy controls scored the highest, followed by the post-operative and pre-operative VS groups, respectively. Kinematic score for the control group was significantly different from those of the pre- and post-operative groups (both *p*-value < 0.001). No significant difference was observed between kinematic scores computed for pre-operative and post-operative timepoints (*p*-value > 0.05). Taken together, this analysis suggests the usefulness of computing a single kinematic score using the head-on-trunk kinematic parameters extracted from the most altered exercises.

## Discussion

In this study we quantified head-on-trunk kinematics to determine if they are altered in individuals with VS during voluntary balance and gait tasks. Head-on-trunk movements were measured in individuals with VS before and then 6 weeks after surgical tumor removal, with vestibular nerve deafferentation. We found that head-on-trunk kinematic measures significantly differed for individuals with VS at both time points compared to healthy controls, particularly for tasks that required controlling balance in tandem position (narrow base of support), on a foam surface (unstable base of support), or without visual input (eyes-closed). Additionally, we found that these same kinematic measures were comparable for individuals with VS before versus after surgery. Thus, our findings indicate that head-on-trunk kinematics are altered in individuals with VS during voluntary balance and gait behaviors. Additionally, our results further suggest that adaptions in the motion strategies of individuals with VS occur prior to surgery and remain abnormal at 6 weeks post-operatively.

Previous studies, using posturographic assessments of standing balance or functional and patient-perceived measures, have shown that individuals with peripheral vestibular loss demonstrate compromised postural stability^30–33^. While impairments could be in part due to the diminished efficacy of the vestibulo-collic reflex^2–4^, to date surprisingly few studies have explored the kinematic interplay of head and trunk in individuals with peripheral vestibular loss^11,12,14,15,34^. Paul et al.^14,15^ quantified head-on-trunk kinematics during gait tasks in vestibular schwannoma. However, in these two studies participants were instructed to perform active yaw head movements during walking, and the subsequent analysis was limited to 1-dimensional yaw motion^14,15^. Indeed, to our knowledge, our current study is the first to investigate head-on-trunk stabilization in all six dimensions of motion during standard voluntary balance and mobility exercises.

A key finding of our present study was that post-operative individuals with unilateral vestibular loss demonstrated altered head-on-trunk kinematics as compared to healthy controls. Specifically, these individuals, who were within the sub-acute stage after their tumor resection (6 weeks post-operative) showed significantly greater range and variability of angular velocity, particularly in the absence of visual information, compared to age-matched controls. These differences were particularly marked during the balance exercises that involved diminished visual and/or somatosensory information (e.g., stance with eyes closed, foam base of support). Analogous results have been recently reported in post-operative individuals with VS for the analysis of 6-dimensional head in space kinematics^35,36^. Indeed, persistent general balance impairments, quantified by measuring the center of pressure while standing on a force plate, remain even in the chronic stages following the VS surgery^37^. Neurophysiological studies in nonhuman primates have, however, established that vestibular reflex pathways actually do demonstrate remarkable compensation in response to vestibular loss^38–41^. Neural mechanisms underlying this compensation include sensory substitution of extra-vestibular self-motion inputs (i.e., proprioceptive and motor related signals) within the first month. Thus, in this context, our present results emphasize the importance of vestibular rehabilitation as well as developing novel rehabilitative interventions that can more effectively recruit such central compensation mechanisms to facilitate more optimal recovery of postural control in individuals with VS.

Additionally, our current results establish that individuals with VS experience altered head-on-trunk kinematics even *before* their surgery, as compared to age-matched controls. This finding is of particular clinical importance since it reveals that head-on-trunk stabilization in pre-operative individuals with VS is impaired, contrary to the prevailing view that central mechanisms can provide virtually complete compensation due to the slowly progressing nature of the VS tumors^11,42,43^. In this context, our present findings contribute to the growing body of work^35,36,44^ suggesting that the gradual adaptation of central mechanisms is not sufficient to provide full compensation. It has been previously addressed that initiating vestibular rehabilitation prior to the VS surgery can help alleviate long-term post-operative symptoms^45,46^. Given our abovementioned finding, we also believe that implementing pre-operative vestibular and balance rehabilitation as parts of best practice guidelines^47^ may enhance postural control, and consequently prevent falls in people awaiting a surgery, particularly in older people who live with VS and are at a higher risk of falling^33^.

We had initially hypothesized that kinematics of head stabilization would show further impairment following the VS surgery compared to before the surgery. This is because it is generally believed that the surgical deafferentation interferes with the previously learned central compensation^31,42,43^. Surprisingly, however, our present findings did not detect any significant differences in head-on-trunk kinematics between the pre-operative and post-operative time points. Instead, head-on-trunk kinematics were similarly altered at both time points (i.e., before and after surgery) in comparison to healthy controls, suggesting that early changes in head stabilization strategy due to presence of the tumor effectively set the upper limit for recovery in individuals with VS. Thus, the tumor resection surgery worsened the head-on-trunk stability acutely, and the compensation that occurred over the next 6 weeks only returned head-on-trunk kinematics patterns to their pre-operative levels. We had initially intended to conduct an additional follow-up assessment 6 months post-surgery. However, due to logistical challenges (many participants were from locations distant from the assessment center) we were unable to collect data for most participants. Nevertheless, based on the Zobeiri et al.^35^ findings that “head” kinematics differ little 6 months versus 6 weeks after VS surgery, we predict there would be little additional change in head-on-trunk kinematics 6-month post-surgery.

We suggest that future work should consider capturing kinematic parameters as soon as possible, and at many time points, in the pre- as well as post-operative stages to (a) identify the trajectories of change in head-on-trunk kinematics over time, and (b) develop new interventions to optimize compensation even before surgery. Moreover, our findings suggest that reduced head stabilization in individuals with VS (demonstrated as higher variability in pre-operative head-on-trunk kinematics) is associated with poorer gait function, greater variability of ipsi-lesional VOR gain, and reduced balance confidence in this population.

Another interesting finding of our study was that the range and variability of rotational velocities specifically differed in the roll and yaw axes for healthy controls versus both pre-operative and post-operative individuals with VS. We speculate that our results regarding change in head-on-trunk stabilization along the roll axis in VS have particular clinical importance. Notably, a recent study of perceptual thresholds in asymptomatic adults aged 21–84 years reported that increased roll tilt thresholds were associated with subclinical postural instability during testing that included standing with eyes closed on unstable surfaces^48^. Furthermore, prior research has established a link between the recovery assessed by DVA and intact otolith (roll) function (determined by vestibular evoked myogenic potential test) in individuals with VS; however, such an improved function was not correlated with canal function^17^. Therefore, in this specific context our present finding in conjunction with these previous studies underscore the importance of emphasizing the evaluation of roll kinematics in individuals with VS. Further research is required to explore the association of impaired roll kinematics with risk of falls in this population.

Lastly, our results demonstrate that it is possible to compute a single kinematic score for head-on-trunk stabilization from only a small subset of our balance exercises. Specifically, we focused on those challenging exercises for which the most significant differences were observed between individuals with VS and healthy controls, namely “tandem stance with eyes closed” and “standing on foam with eyes closed”. This result extends the results of a previous study from our lab^36^ in which we reported that a single kinematic value computed from the same subset of exercises was remarkably different between the healthy controls and individuals with VS. Overall, our present kinematic score shows that head-on-trunk kinematics are as informative as head-in-space kinematics in terms of distinguishing healthy controls from individuals with VS at pre- and post-operative time points. This emphasizes the general significance of both head and trunk kinematics in balance dysfunction.

### Limitations

Small sample size was a main limitation in this study. Although we had originally planned to conduct pre-operative and post-operative assessments with 18 VS participants, due to distance and time constraints, 9 participants were unable to travel to our clinic to complete their 6 weeks post-operative tests. Additionally, our sample had a relatively wide age range, therefore, our results might have been biased to some extent by the effect of aging on balance control; however, to minimize this effect we included a healthy age-matched control group. Finally, all participants with VS in this study received suboccipital craniotomy surgery; therefore, our results might not be applicable to those undergone other surgical approaches.

## Conclusions and implications

Here we have shown that head-on-trunk movements are altered in individuals with VS even prior to surgery, due to the presence of the tumor. Differences were particularly pronounced in situations where alternative extra-vestibular sensory information was not present, indicating that central compensation and/or post-operative re-adaptation is not sufficient to account for the altered motor control in this group of individuals. Taken together our findings suggest that early changes in sensorimotor strategies set the upper limit for recovery in these individuals resulting in incomplete compensation that may warrant pre-operative rehabilitation.

### Supplementary Information

Supplementary Information.

## Data Availability

The datasets used and analyzed in the present study are available from the corresponding author on reasonable request.
