# Supplementary material for: Effect of vestibular loss on head-on-trunk stability in individuals with vestibular schwannoma
Source: Sci Rep. 2024 Feb 12;14:3512. doi: 10.1038/s41598-024-53512-3 (PMC10861475; doi:10.1038/s41598-024-53512-3)
Supplement: Supplementary file 1 — Supplementary Information. [file 41598_2024_53512_MOESM1_ESM.pdf]

Effect of vestibular loss on head-on-trunk stability in individuals with vestibular schwannoma

Raabeae Aryan[1], Omid A. Zobeiri[1,2], Jennifer L. Millar[3], Michael C. Schubert[3,4], Kathleen E. Cullen[1,2,4,5,6,*]

Running title: Head-on-Trunk Kinematics in Vestibular Loss

Author Affiliations:
[1]Department of Biomedical Engineering, Johns Hopkins University, Baltimore, MD, USA
[2]Department of Biomedical Engineering, McGill University, Montreal, QC, Canada
[3]Department of Physical Medicine and Rehabilitation, Johns Hopkins University School of Medicine, Baltimore, MD, USA
[4]Department of Otolaryngology-Head and Neck Surgery, Johns Hopkins University School of Medicine, Baltimore, USA
[5]Department of Neuroscience, Johns Hopkins University School of Medicine, Baltimore, USA
[6]Kavli Neuroscience Discovery Institute, Johns Hopkins University, Baltimore, MD, USA

***Corresponding Author:**
Kathleen E. Cullen, Johns Hopkins University School of Medicine
720 Rutland Ave, Traylor 504, Baltimore, MD 21205-2109, USA.
Email: kathleen.cullen@jhu.edu

Supplemental Figure S1

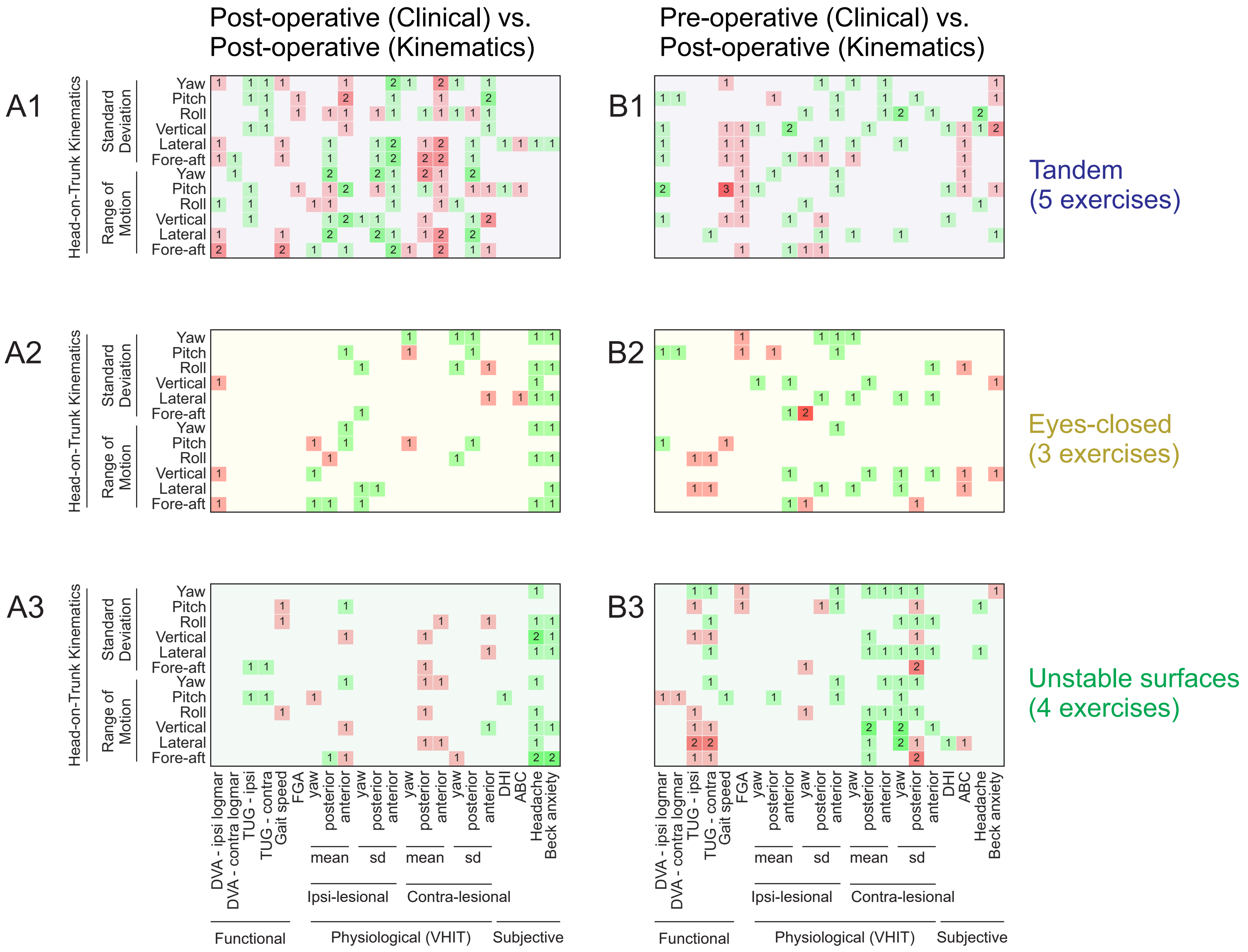

Figure S1: This figure illustrates the results of correlational analysis between the post-operative head-on-trunk kinematics and post-operative clinical measures (Fig. S1.A) or pre-operative clinical measures (Fig. S1.B). Figures S1.A illustrates significant correlations between post-operative head-on-trunk kinematics and post-operative clinical measures. Notably, multiple post-operative head-on-trunk kinematic measures demonstrated significant correlations with post-operative functional and physiological measures; however, these correlations were not consistent across most tasks (i.e., <50% of tasks). Lastly, we investigated the relationships between post-operative head-on-trunk kinematic measures and pre-operative clinical measures as shown in Figures S1.B. We found that post-operative head-on-trunk pitch range of motion was negatively correlated with gait speed during most tasks in tandem position (Fig. S1.B1). In tasks performed with eyes-closed (Fig. S1.B2) a consistent correlation between the kinematic measures and clinical measures was only observed between SD of ipsi-lesional horizontal VOR (yaw vHIT) and the SD of fore-aft head-on-trunk kinematic measure (2/3 tasks). No consistent correlation between the kinematic measures and clinical measures was found across tasks that required standing on unstable surface (Fig. S1.B3). Thus, pre-operative functional and physiological clinical kinematic measures were correlated with kinematic measures mainly during controlling balance in tandem position or with eyes closed.

Supplemental Tables

Table S1: Standard deviations of head-on-trunk motion in each of the 6 axes for healthy controls as well as pre-operative and post-operative VS subjects (mean±SD)

	**Linear acceleration (mG)**			**Angular Velocity (deg/s)**		
**Tasks**	Fore-aft	Lateral	Vertical	Roll	Pitch	Yaw
				**Healthy Controls**		
Tandem walk forward (FGA#7)	64.5±18.7	70.4±29.2	71.6±30.3	8.6±3.1	14.0±8.8	11.2±2.1
Tandem walk forward	74.5±40.3	78.7±41.8	79.9±45.9	9.4±3.6	14.8±15.7	12.2±4.3
Tandem walk backward	83.8±45.2	79.4±33.0	75.9±45.0	8.7±4.4	16.9±21.2	12.3±5.4
Standing on firm, eyes closed	19.5±4.2	16.2±4.6	11.5±4.9	0.6±0.2	0.96±0.4	0.9±0.4
Standing on foam, eyes open	18.8±6.8	16.7±4.5	12.6±5.5	0.99±0.4	1.1±0.6	1.2±0.4
Standing on foam, eyes closed	23.5±6.3	25.1±6.1	17.1±7.1	1.9±0.9	2.1±1.4	1.9±0.5
Tandem stance, eyes open	23.8±18.2	17.5±6.8	15.4±8.3	0.9±0.4	1.4±0.9	1.7±0.4
Tandem stance, eyes closed	24.9±8.2	25.8±7.5	21.6±8.1	1.8±0.7	2.5±1.0	3.3±1.3
Foam cup balance 1 foot	34.8±12.8	44.2±19.2	23.4±5.5	2.4±1.0	3.6±1.6	3.9±1.7
Foam cup alternatively foot	71.7±34.8	88.4±34.4	60.9±37.3	9.1±3.3	17.3±18.7	9.7±3.8
				**Pre-operative VS**		
Tandem walk forward (FGA#7)	98.1±26.7	112±28.2	96.1±23.7	11.7±3.9	22.6±12.2	16.0±3.0
Tandem walk forward	108±36.9	109±31.2	94.1±26.8	11.4±2.4	24.2±13.2	15.2±2.9
Tandem walk backward	99.8±25.0	99.9±27.8	95.4±33.0	10.9±2.8	24.5±14.6	13.6±3.0
Standing on firm, eyes closed	24.1±10.9	18.7±11.8	19.9±13.0	1.3±1.1	2.8±3.1	1.9±1.8
Standing on foam, eyes open	20.3±6.7	18.4±6.9	16.5±5.19	1.7±0.8	2.2±1.1	1.9±1.0
Standing on foam, eyes closed	40.2±19.2	38.2±8.6	43.5±36.8	3.8±1.3	6.5±7.0	4.2±1.9
Tandem stance, eyes open	30.5±15.5	28.5±17.1	26.2±16.0	2.8±1.8	3.8±2.6	3.9±1.8
Tandem stance, eyes closed	56.5±26.3	68.8±35.1	51.6±22.0	6.0±2.2	9.6±5.9	9.3±4.4
Foam cup balance 1 foot	61.9±40.7	67.2±35.4	44.6±28.6	5.2±2.8	14.4±17.5	6.9±2.9
Foam cup alternatively foot	74.1±25.4	98.7±25.4	63.3±21.3	9.4±2.1	17.1±9.2	10.7±2.2
				**Post-operative VS**		
Tandem walk forward (FGA#7)	102±34.5	124±40.8	103±33.0	12.0±3.9	16.6±9.4	16.8±4.6
Tandem walk forward	125±57.0	124±36.0	105±22.8	12.1±1.4	18.5±6.3	17.1±3.6
Tandem walk backward	114±30.7	117±26.6	102±19.1	10.8±0.9	18.3±7.5	15.9±2.3
Standing on firm, eyes closed	20.9±10.2	19.0±7.7	17.1±11.6	1.34±0.91	2.4±1.9	2.1±1.1
Standing on foam, eyes open	22.6±22.5	19.4±7.6	16.5±8.5	1.6±0.8	1.95±1.4	1.96±0.9
Standing on foam, eyes closed	42.8±27.2	49.0±14.0	42.1±21.3	4.4±1.1	5.5±2.9	5.3±1.9
Tandem stance, eyes open	25.8±14.7	28.9±13.3	26.4±12.5	2.6±1.5	3.6±3.1	4.2±1.9
Tandem stance, eyes closed	48.9±37.6	68.6±48.6	58.5±49.6	6.2±4.9	7.1±5.0	7.96±3.3
Foam cup balance 1 foot	50.0±35.8	61.2±38.2	42.8±23.9	4.8±2.9	8.97±8.6	7.2±3.5
Foam cup alternatively foot	70.1±23.4	103±32.1	63.2±10.6	9.4±2.8	12.9±2.6	11.3±2.4

VS: vestibular schwannoma. FGA: Functional Gait Assessment.

Table S2: Demographics of participants with vestibular schwannoma.

Subjects	Sex	Age (y/o)	Height (m)	Weight (kg)	Lesion location	Surgery type	Tumor volume (cm^3)
1	M	23	1.88	131	Right	SOC	1.5 x 1.0 x 1.1
2	M	62	1.77	85.5	Right	SOC	2.0 x 2.3 x 2.1
3	M	41	1.80	102.6	Left	SOC	2.2 x 2.2 x 2.5
4	M	49	1.82	86.1	Right	SOC	4.3 x 3.1 x 3.7
5	M	64	1.85	116.9	Left	SOC	0.8 x 0.6 x 0.7
6	M	63	1.70	81.6	Right	SOC	1.9 x 1.2 x 1.1
7	M	70	1.78	74.8	Left	SOC	2.2 x 1.4 x 1.3
8	M	57	1.70	94.8	Right	SOC	1.6 x 1.3 x 1.1
9	M	72	1.78	95.5	Right	SOC	0.5 x 0.6 x 1.2

M: male. SOC: Suboccipital Craniotomy surgery.

Table S3: Pre-operative functional behavioral, physiological, and dizziness characteristics of individuals with vestibular schwannoma.

Subjects	DVA Static logMar	*DVA Ipsi logMar	*DVA Contra logMar	vHIT Contra Yaw	vHIT Ipsi Yaw	vHIT Contra Ant	vHIT Ipsi Ant	vHIT Contra Post	vHIT Ipsi Post	DHI
1	-0.14	0.12	0.1	0.91	0.82	0.74	0.93	0.95	0.72	0
2	0	0.22	0.24	1.07	1.16	0.49	0.37	0.89	0.52	48
3	-0.2	0.24	0.28	0.8	0.61	0.95	0.29	0.75	1.15	16
4	-0.08	0.84	0.58	1	0.46	0.66	0.34	0.5	0.51	40
5	-0.2	0.3	0.32	1.01	1.02	0.92	0.97	0.86	0.76	0
6	-0.04	0.4	0.38	0.81	0.36	0.37	0.58	0.58	0.32	22
7	0.26	0.32	0.22	0.55	0.86	0.65	0.7	0.71	0.55	2
8	0.18	0.3	0.24	0.89	0.85	0.45	0.77	0.73	0.67	2
9	-0.14	0.52	0.38	1.06	1	0.91	0.88	1.04	0.41	54

Ipsi: Ipsi-lesional side. Contra: contra-lesional side. DVA: Dynamic Visual Acuity. vHIT: Video Head Impulse Test (VOR gain). Ant: Anterior. Post: Posterior. DHI: Dizziness Handicap Inventory. *DVA is corrected to account for static visual acuity.
